# Valorization of Blueberry Processing Byproducts for the Development of Intelligent Packaging: A Comparative Study on Biomass versus Extract Use in pH-Sensitive Films

**DOI:** 10.1021/acsmaterialsau.5c00150

**Published:** 2025-10-27

**Authors:** Milica Arizanova, Elena Velickova, Darko Dimitrovski, Danila Merino

**Affiliations:** † Department of Food Technology and Biotechnology, Faculty of Technology and Metallurgy, 63579 Ss Cyril and Methodius University in Skopje, Rugjer Boskovic 16, 1000 Skopje, North Macedonia; ‡ POLYMAT, Basque Center for Macromolecular Design and Engineering, University of the Basque Country UPV/EHU, Avenida de Tolosa 72, 20018 Donostia-San Sebastian, Spain; § Ikerbasque, Basque Foundation for Science, 48009 Bilbao, Spain

**Keywords:** chitosan, bioplastics, blueberry pomace, food waste, biomass, pH indicator, food spoilage

## Abstract

The simultaneous challenges of plastic pollution and food waste call for sustainable materials that valorize agro-industrial residues. In this study, multifunctional chitosan-based films were developed by incorporating up to 30 wt % of mildly hydrolyzed blueberry pomace (BBP), a juice industry byproduct rich in anthocyanins and fibers. Unlike conventional approaches that rely on anthocyanin extracts, our full-biomass strategy integrates both soluble and insoluble pomace fractions directly into the chitosan matrix and benchmarks these against extract-only controls. The resulting films exhibited enhanced opacity, UV-blocking ability, and strong pH-responsive color changes—attributes critical for intelligent food packaging. FTIR and SEM analyses confirmed strong interactions between BBP and chitosan, while mechanical tests revealed increased flexibility and moderate reinforcement from BBP fibers. Although both BBP and extract additions slightly reduced thermal stability and increased water solubility, they did not compromise barrier properties; BBP-loaded films maintained water vapor permeability, while extract-loaded films showed a minor increase. Importantly, BBP-containing films showed superior pH responsiveness and sustained antioxidant release over time, highlighting their multifunctionality. This work demonstrates a scalable, zero-waste packaging strategy aligned with circular economy principles through the upcycling of crustacean shell waste and fruit pomace into high-performance smart films.

## Graphical abstract

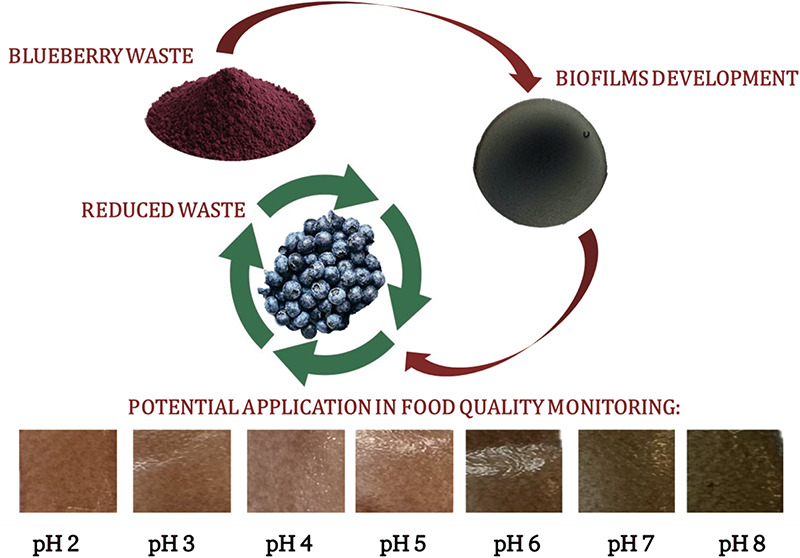

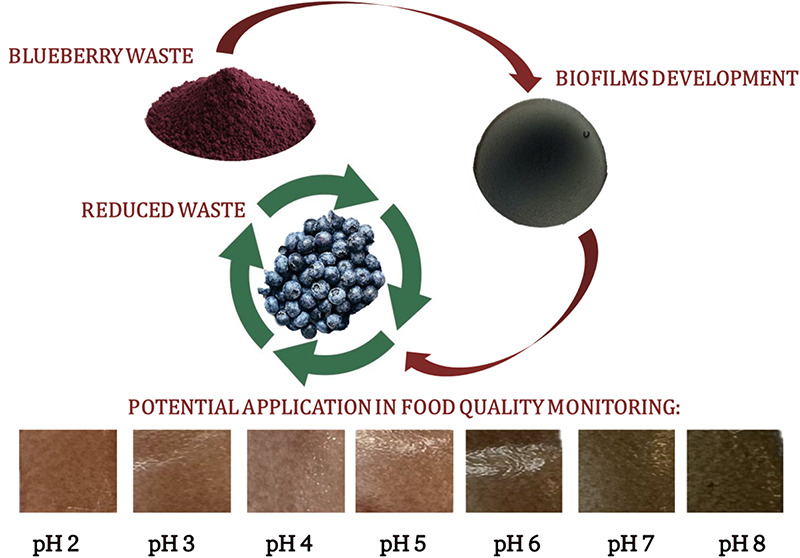

## 1 Introduction

Food loss and waste, together with plastic pollution from food packaging, have become pressing environmental issues, driving both resource inefficiency and ecosystem degradation. Food loss (the reduction in edible food occurring along the supply chain from harvesting through processing and distribution) and food waste (the discarded of safe, edible food at the consumer level), together represent nearly one-third of all food produced globally.^1–4^ Valorizing these losses and their byproducts not only curbs waste but also offers sustainable alternatives to petrochemical plastics.^5^ According to the FAO, food loss and waste in the fruit and vegetable industries alone account for 60% of total production.^1,6,7^ A large fraction of this (peels, seeds, and pomace discarded during juice processing) remains underexploited despite their high nutritional value, functional compounds, and polymer hydrocolloid content. Left untreated, these residues contribute to environmental contamination owing to their high moisture content and microbial load.^8^ In recent years, upcycling these byproducts into biodegradable polymer composites has emerged as a promising route to both reduce plastic use and enable real-time food-quality monitoring.^2,9–11^

Blueberry pomace (BBP), a residue from juice and puree production, is exceptionally valuable due to its high anthocyanin content.^12,13^ These water-soluble pigments not only provide antioxidant benefits but also exhibit pronounced color changes across pH ranges, making them ideal for intelligent packaging applications.^14^ Anthocyanin concentrations in dried BBP (≈125.82 ± 5.89 mg cyanidin-3-glucoside equivalents per 100 g dry weight) often exceed those in whole berries (25–495 mg/100 g fresh weight), due to the skin-rich nature of the pomace.^15^ BBP has been previously valorized in the development of functional foods such as cookies^15,16^ and fermented dairy beverages,^17^ and more recently in composite packaging materials. In most cases, BBP-derived anthocyanin extracts, typically obtained via ethanolic^18,19^ or aqueous extraction,^20^ have been incorporated into biopolymers to impart pH-responsive color changes. However, such extraction processes generate secondary biomass waste and limit the circularity of the system. A few studies have attempted to incorporate whole BBP into starch- or starch–chitosan-based films, but these often showed limited color shifts and only under direct immersion conditions.^21,22^ Moreover, the loading of BBP was generally restricted to ≤15 wt % due to its detrimental impact on mechanical performance.^22,23^

Chitosan (Ch), a biopolymer derived from the deacetylation of chitin, is obtained from crustacean shell waste (e.g., shrimp, crab, lobster). This biopolymer is well-known for its excellent film-forming ability, biodegradability, and intrinsic antimicrobial activity against a broad spectrum of bacteria, including both Gram-positive and Gram-negative strains.^24–28^ Chemically, chitosan is a linear polysaccharide composed mainly of β-(1→4)-linked d-glucosamine and N-acetyl-d-glucosamine units.^29^ Its mechanical performance can be tailored with the addition of plasticizers like glycerol for flexibility and emulsifiers like Polysorbate 80, for enhanced cohesion and handling.^30,31^

The objective of this work is to develop multifunctional and sustainable chitosan-based films incorporating up to 30 wt % of mildly hydrolyzed blueberry pomace (BBP) aiming to valorize two agro-industrial byproducts, crustacean shell waste and fruit pomace, within a single packaging system. Hydrolysis of BBP was performed to disrupt plant cell walls and partially depolymerize polysaccharides, thereby facilitating the release of anthocyanin.^32^ Beyond reducing waste, our strategy targets enhanced film performance for food packaging applications by leveraging the pH-responsive color change and antioxidant capacity of anthocyanins, while also introducing natural antimicrobial functionality from chitosan.

## 2 Materials and Methods

### 2.1 Materials

Organic wild BBP was purchased from LOOV Organic LLC (Estonia). Its specification is presented in Figure S1. Medium molecular weight chitosan (190 000 – 310 000 Da) with a degree of deacetylation of >75% was purchased from Merck (Italy). Glycerol and polysorbate 80, used here as plasticizers, were purchased from Thermo Scientific (Germany). The acetic acid was purchased from Merck (Italy). All chemicals were of analytical grade.

### 2.2 Biomass Mild Acidic Hydrolysis

Before incorporating the biomass into the production of biopolymer films, BBP was hydrolyzed following the method described by Merino et al.^33^ Briefly, BBP was dispersed at 5 wt % in 1 M acetic acid and stirred continuously at 40 °C for 24 h.

### 2.3 Biomass and Extract Preparation

Following hydrolysis, the biomass was divided in two fractions: one portion was incorporated as a filler in Bioplastic preparation, while the other was filtered through a 0.45 μm nylon membrane to obtain an extract with a final concentration of 1.67 wt % determined gravimetrically.

### 2.4 Chitosan Composite Films Preparation

A 2% (w/v) Ch solution was prepared by dissolving medium-molecular-weight Ch in 2% (v/v) aqueous acetic acid under constant stirring. Glycerol and polysorbate 80 were then added at 20% (w/w) each relative to Ch to act as plasticizers. Hydrolyzed blueberry pomace biomass (B) or its filtered anthocyanin extract (E) was incorporated into this film-forming solution at loadings of 10, 20 and 30% (w/w) and homogenized for 30 min to ensure uniform dispersion. Film formulations and labels are presented in Table S1. All films were prepared by casting the film forming solutions into plastic Petri dishes with a diameter of 13 cm (Figure 1). The solvent was evaporated under a fume hood and after drying, films were peeled off and stored in a ventilated chamber at 50% relative humidity (RH) and 25 °C prior to analysis.

**Figure 1:**
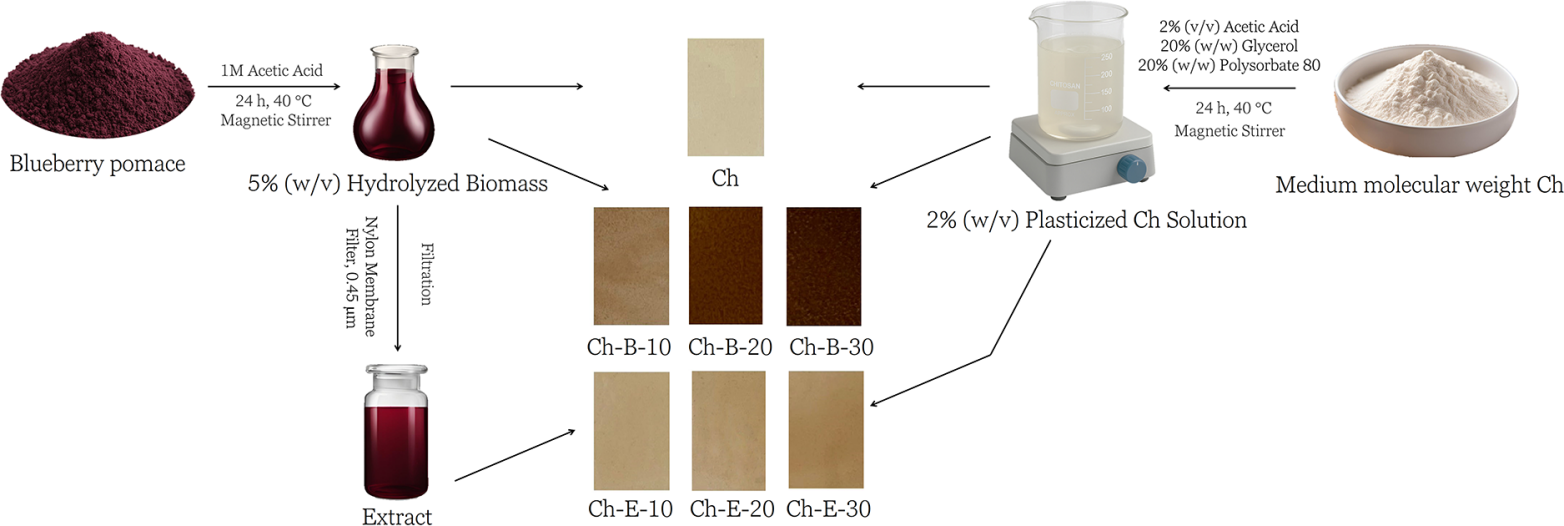
Schematic representation of the film preparation process.

### 2.5 Film Characterization

#### 2.5.1 Film Thickness (δ)

The film thickness was measured using a digital caliper with accuracy of ±0.02 mm (Mitutoyo, USA). Ten measurements were taken at different locations across each film sample, and the mean thickness value was used for all subsequent calculations.

#### 2.5.2 Scanning Electron Microscopy (SEM)

The surface and cross-section morphology of the composite Bioplastic films were examined using a Hitachi TM3030Plus (Tokyo, Japan) scanning electron microscope. To prepare cross-section samples, the films were first cooled in liquid nitrogen and then fractured to expose their cross-sectional area. Prior to imaging, all samples were coated with a thin sputtered gold layer to enhance conductivity. Imaging was performed at an accelerating voltage of 15 kV and a working distance of 10 mm, using 300× magnification for surface views and 2000× for cross-sectional micrographs.

#### 2.5.3 Fourier Transform Infrared Spectroscopy (FTIR)

FTIR spectra of the BBP, B, E and the seven film formulations were determined with a Thermo Scientific Nicolet iS20 FTIR spectrometer (Massachusetts, USA). Data collection was performed in the 4000–600 cm^–1^ range with a 4 cm^–1^ spectral resolution and the total number of scans was 32.

#### 2.5.4 Mechanical Properties

The mechanical properties of the films were assessed using a Texture Analyzer TA-HD plus (Surrey, UK). Dog-bone-shape specimens were equilibrated at 25 °C and 50% relative humidity for 3 days prior to testing. At least eight replicates per formulation were mounted in tensile grips and stretched at a constant speed of 0.42 mm/s until failure. From the resulting stress–strain curves, Young’s modulus, tensile strength, and elongation at break were calculated.

#### 2.5.5 Thermogravimetric Analysis (TGA)

The thermogravimetric tests were carried out using a thermal analyzer TGA8000 Perkin Elemer (Massachusetts, USA). Samples were heated at a constant rate of 10 °C/min from 40 to 800 °C under nitrogen atmosphere. The initial weight of the biomass, extract and film samples were ∼5 mg.

#### 2.5.6 Differential Scanning Calorimetry (DSC)

DSC analysis was performed using TA DSC 2500 (New Castle, Delaware, USA). Approximately 5–10 mg of each film sample was sealed in an aluminum pan and measured under a nitrogen purge. Samples were heated from −20 to 120 °C at 10 °C/min, cooled to −20 °C, and then reheated at the same rate. Glass transition temperatures (*T*_g_) were determined from the second heating ramp.

#### 2.5.7 Moisture Content (MC)

MC of the films was determined by weighing pieces of ∼0.4 g of each formulation before and after drying in a vacuum oven at 40 °C for 24 h. Three measurements for each formulation were performed. Moisture content was calculated as

$$\mathrm{MC}(\%)=\left(\frac{m_{i}-m_{f}}{m_{i}}\right)\times 100\%$$

where *m*_*i*_ is the initial weight and *m*_*f*_ is the dry weight of the samples.

#### 2.5.8 Water Solubility (WS) and Degree of Swelling (DS)

WS of the films was determined according to the methodology proposed by Quilez-Molina et al.^34^ In order to determine the initial dry matter of the films (*m*_*i*_), samples with 2 cm diameter were dried in a vacuum oven at 40 °C for 6 h. Each sample was immersed in 20 mL of distilled water, at room temperature for 24 h, with periodical stirring. Then, the sample was removed from water, dried at 40 °C for 6 h, and weighted to determine the final mass (*m*_*f*_). The film solubility (%) was calculated using the following equation:

$$\mathrm{WS}(\%)=\frac{m_{i}-m_{f}}{m_{i}}\times 100\%$$

The average value was calculated as the mean of three measurements for each film formulation and results are reported as mean ± standard deviation (SD).

The degree of swelling (%) was conducted by immersing the dried films (*m*_*i*_) into 20 mL distilled water for 24 h at room temperature, according to the methodology proposed by Velickova et al.,^35^ with some modifications. The swollen samples were removed, blotted with tissue paper so the excess water is removed and the 24 h-swollen weight of the films was noted (*m*_*e*_). The degree of swelling was calculated using the following equation:

$$\mathrm{DS}(\%)=\frac{m_{i}-m_{e}}{m_{i}}\times 100\%$$

#### 2.5.9 Water Vapor Permeability (WVP)

The water vapor permeability of the films was determined using TQC Sheen permeability cups (Capelle aan den IJssel, The Netherlands), with an exposed surface area of 10 cm^2^. The cups were filled with 4 mL distilled water to simulate a 100% RH and each film sample was fixated on top of the cup using the seal ring of the permeability cup. The cup was then placed into a dry chamber containing dry silica gel beads. The water vapor permeability test was carried out at 20 °C and RH 20%, with periodically weighing the samples for 6 h. The change of the weight determines the rate of vapor movement through the films from the distilled water to the controlled atmosphere. The weight loss of the capsules was plotted and linearized and the slope of the resulting graph (g/s) was then divided by the exposed film surface area (0.001 m^2^) to calculate the water vapor transmission rate (WVTR):

$$\mathrm{WVTR}=\frac{\Delta m}{t\times A}$$

where Δ*m* is the weight loss of the capsules (g), *t* is time (s) and *A* is the exposed film surface area (m^2^).

This value was used to calculate the WVP using the following equation:

$$\mathrm{WVP}=\frac{\mathrm{WVTR}\times \delta }{P_{H_{2}O}\times \Delta \mathrm{RH}}$$

where δ is the average film thickness, $P_{H_{2}O}$ is the water vapor saturation pressure at the test temperature, and ΔRH is the difference in vapor pressure through the film.

Three replicates for each formulation were analyzed and the average value was calculated as the mean of three measurements.

#### 2.5.10 Optical Properties

The films’ light transmittance and opacity were measured using a Shimadzu UV-2550 Spectrophotometer (Germany). The films were cut into rectangular strips (1 cm × 4 cm) and placed into the UV–vis chamber, previously calibrated for 100% transmittance with air. Transmittance spectra were recorded from 200 to 800 nm. Opacity was calculated at 600 nm according to Li et al.,^36^ using the following equation:

$$\mathrm{Opacity}=\frac{-\log\;T_{600}}{\delta }$$

where *T*_600_ is the light transmittance at a wavelength of 600 nm, and δ is the film thickness in mm.

#### 2.5.11 Color Analysis

The color change of the films according to different pH was monitored. The films were cut into rectangular strips (1 cm × 4 cm) and immersed in pH buffers ranging from 2 to 8 (citrate buffer (pH 2–3), acetate buffer (pH 4–5) and phosphate buffer (pH 6–8)) for 10 min. Then, they were blotted with tissue paper so the excess buffer is removed and their color was photographed. The colorimetric parameters L*, a*, and b*—corresponding to lightness, red-green, and yellow-blue values, respectively—were recorded after 24 h of drying, using consistent lighting and distance conditions. Measurements were taken with a free mobile application called “Colour Picker.” These values were then used to calculate the total color difference (Δ*E*) relative to the control sample, as shown in the equation:

$$\Delta E=[(L-L^{*}{)}^{2}+(a-a^{*}{)}^{2}+(b-b^{*}{)}^{2}{]}^{0.5}$$

where L*, a*, and b* are the colorimetric parameters of the pristine sample.

#### 2.5.12 Antioxidant Properties

The antioxidant capacity of the biocomposites was evaluated against the DPPH• radical, following the methodology reported by Merino et al.^37^ Discs of 16 mm diameter (approximately 0.05 g) of the film samples were added to 4 mL of 0.1 mM DPPH solution in ethanol. The decrease in the absorbance of the solution due to the antioxidant films’ action was monitored at 517 nm for 24 h using a Cary 6000i UV–vis–NIR Spectrophotometer (USA). Subsequently, the radical scavenging activity (RSA) was determined using the following equation:

$$\mathrm{Radical}\;\mathrm{scavenging}\;\mathrm{activity}(\%)=\frac{A_{0}-A_{1,t}}{A_{0}}\times 100$$

where *A*_0_ is the absorbance at 517 nm of the DPPH• radical solution at the beginning of the measurement, and *A*_1,*t*_ is the absorbance at 517 nm of the radical solution with the sample, measured at different times.

#### 2.5.13 Statistical Analysis

All methods were performed in triplicate unless otherwise stated, and results are reported as mean ± standard deviation. Statistical differences were evaluated using one-way analysis of variance (ANOVA) and Tukey’s multiple comparison tests. In all cases, a *p* value of <0.05 was considered significant.

## 3 Results and Discussion

### 3.1 Physicochemical Properties of the Biocomposite Films

All chitosan films, that is neat, biomass-loaded (Ch-B-10/20/30), and extract-loaded (Ch-E-10/20/30) films formed uniform, crack-free sheets with a smooth surface (Figure 2A). Biomass-containing samples appeared clearly more opaque and colored than both the control and extract-only films. Despite these visual differences, film thickness remained essentially unchanged across most formulations. Average thickness values ranged from 0.09 to 0.11 mm, with no statistically significant differences compared to the control, except for the Ch-B-30 sample, which showed a small but significant decrease (0.10 mm vs 0.11 mm for the control; *p* < 0.05) (Figure 2A). This slight decrease can be attributed to the formulation method: in the Ch-B-30 sample, 30% of the chitosan solution was replaced by the blueberry biomass suspension, reducing the overall amount of chitosan, the primary film-forming component in the casting mixture. Consequently, less polymer matrix was available to build up the film structure, resulting in a thinner layer after drying. Additionally, the presence of insoluble biomass particles may have promoted denser packing and lower water retention during film formation, further contributing to the observed reduction in thickness. Kurek et al.^19^ also obtained similar results, that is, the addition of blueberry biomass contributed to a slight reduction in the thickness of the films, while Dordevic et al.^38^ observed no change in thickness with the addition of the extract.

**Figure 2:**
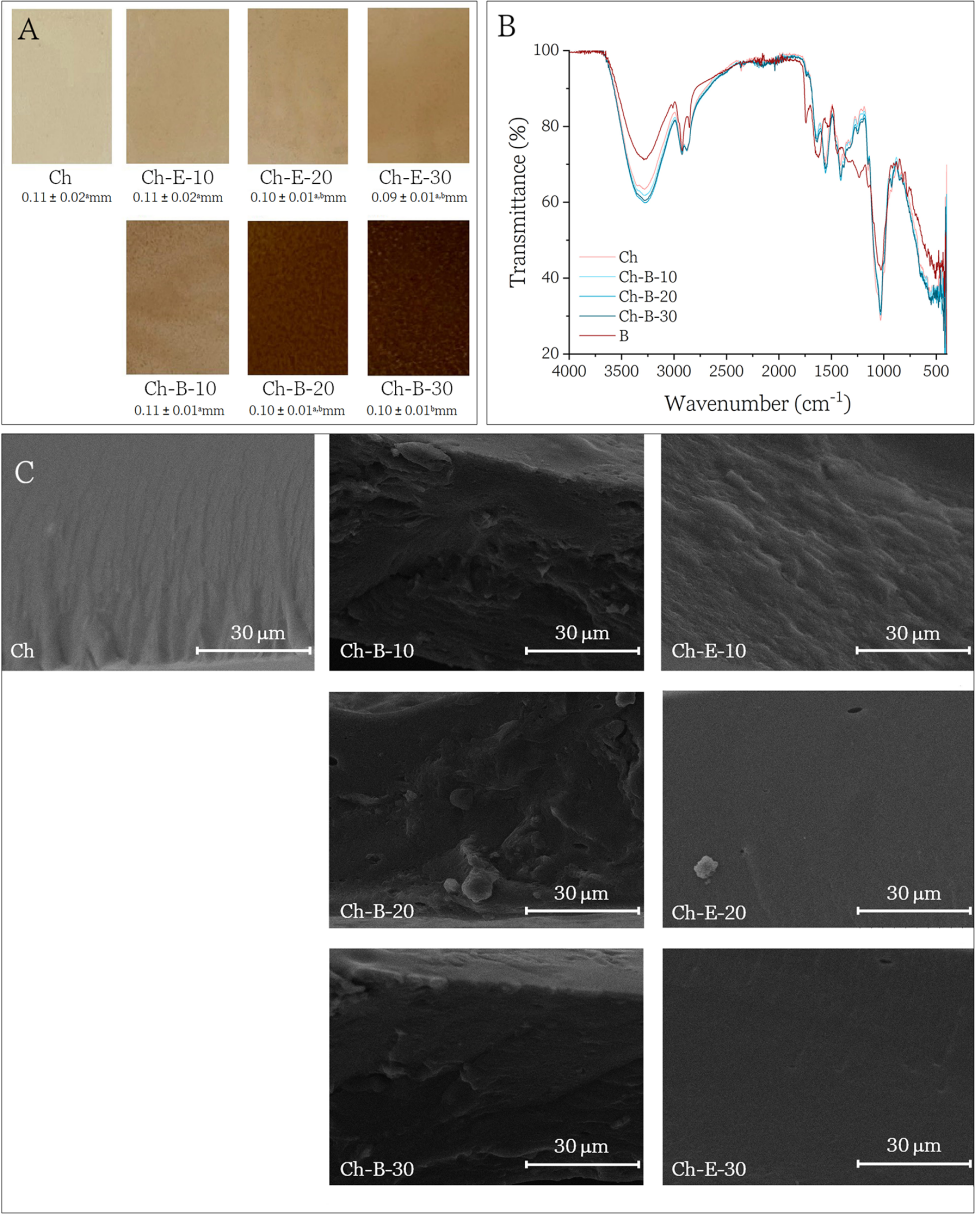
(A) Appearance of the dried films and their thickness (mm); (B) FTIR spectra of Ch, Ch-B-10, Ch-B-20, Ch-B-30 and B; and (C) SEM images from cross-section of Ch, Ch-B-10, Ch-B-20, Ch-B-30, Ch-E-10, Ch-E-20 and Ch-E-30.

FTIR spectra presented in Figure 2B provide insight into the chemical interactions between Ch and B in the composite films. All films exhibit a broad absorption band in the 3450–3100 cm^–1^ region, corresponding to overlapping −OH and −NH stretching vibrations typically associated with intra- and intermolecular hydrogen bonding in chitosan.^37^ In the Ch-B films, this band shows a slight shift toward lower wavenumbers and an increase in intensity compared to the spectra of pure chitosan and B, suggesting the formation of additional hydrogen bonds between B and hydroxyl- and amine-containing groups present in the chitosan polysaccharide.^39^ These spectral changes confirm molecular-level interactions between the two components, indicative of a well-integrated biocomposite matrix.

At 1580–1560 cm^–1^ the corresponding peak of the bending of N–H of chitosan can be seen. In the composite films (Ch-B-10, Ch-B-20, Ch-B-30), this peak shows a progressive decrease in intensity and broadening, indicating the dilution effect of adding B and interactions between chitosan’s amine groups and the phenolic compounds in the blueberry biomass, respectively. In the B sample, a weaker signal is present, possibly due to C=C stretching from aromatic rings in anthocyanins.

A distinct band around 1020 cm^–1^ was observed in all samples, corresponding to C–O–C stretching vibrations associated with glycosidic bonds, characteristic of polysaccharide structures.^40^ This signal is expected, given that chitosan is a polysaccharide and the hydrolyzed blueberry pomace biomass contains a significant carbohydrate fraction (66%, as reported by the supplier), including pectin, cellulose, and hemicellulose.^41^ The subtle differences in peak shape and intensity among the samples likely reflect compositional variations and structural differences between the chitosan backbone and the plant-derived polysaccharides present in B.^42^

Similarly, Figure S2 includes the FTIR of Ch, E, and composites with extract. The FTIR spectra revealed successful incorporation of E into the Ch matrix. In the Ch-E films, the O–H and N–H overlapped band centered at around 3300 cm^–1^ is slightly broader and less intense compared to Ch, suggesting hydrogen bonding between the extract and chitosan. The 1640–1550 cm^–1^ region shows a clear shift to lower wavenumbers in Ch-E films compared to Ch, indicative of interactions between chitosan’s amine groups and phenolic/aromatic compounds in the extract. In the 1400–1000 cm^–1^ region, shifts and changes in band profiles in Ch-E films further confirm the integration of extract components into the film structure.^38^

SEM micrographs of the film surfaces (Figure S3) and cross sections (Figure 2C) revealed generally smooth and compact morphologies across all formulations, suggesting good miscibility and compatibility between chitosan and the incorporated components. Particularly uniform and homogeneous surfaces were observed in the control Ch and Ch-E films, consistent with the absence of insoluble residues. In contrast, the surface and cross sections of films containing Ch-B exhibited slight roughness and irregularities, attributed to the presence of residual fiber fragments not fully solubilized during hydrolysis. Comparable increases in cross-sectional rugosity have been reported in pectin-based films enriched with hydrolyzed lemon peel, where the accumulation of insoluble particles disrupted matrix continuity and introduced microstructural heterogeneity.^43^

To evaluate the impact of the addition of B and E on the mechanical performance of Ch-based films, Young’s Modulus (MPa), tensile strength (MPa), and elongation at break (%) were determined, as shown in Figure 3.

**Figure 3:**
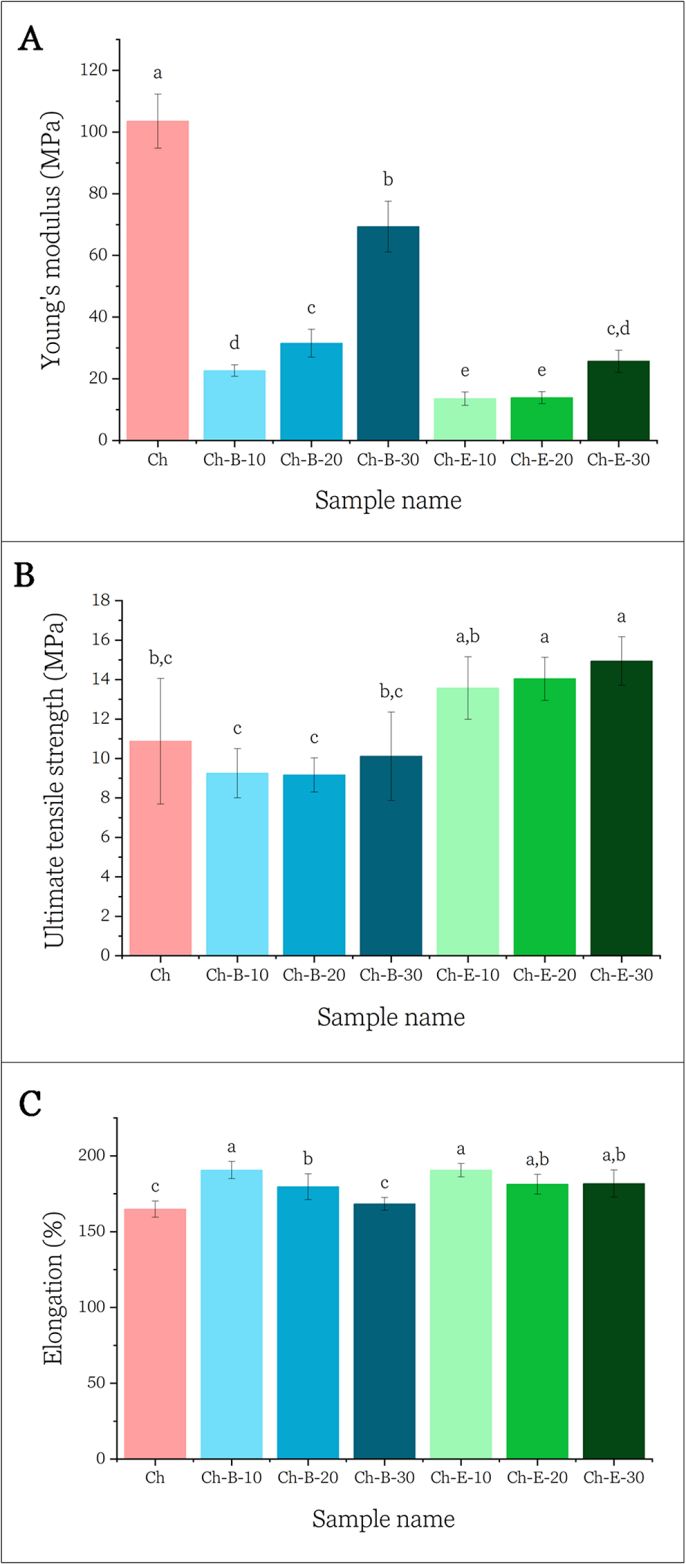
Mechanical properties of chitosan-based films containing hydrolyzed blueberry pomace (Ch-B) or extract (Ch-E) at different loading levels (10–30 wt %). (A) Young’s modulus (MPa), (B) ultimate tensile strength (MPa), and (C) elongation at break (%). Different letters indicate statistically significant differences (*p* < 0.05) according to Tukey’s test.

Overall, the incorporation of both B and E significantly influenced the films’ mechanical behavior. Young’s Modulus (YM), a measurement of the film stiffness, decreased with the addition of both hydrolyzed biomass and extract, although a slight increase was observed at 20 or 30 wt % loading levels. Nonetheless, all composite films exhibited lower YM values than the control chitosan film (Figure 3A). This reduction in stiffness is attributed to the presence of low molecular weight components in B and E, such as sugars, phenolics, and soluble carbohydrates, which interfere with the dense hydrogen bond network of chitosan, leading to a more flexible and less rigid matrix.^44^ Interestingly, films containing B exhibited higher YM values than those with E, likely due to the reinforcement effect of residual insoluble cellulose fibers in the pomace, that contribute to mechanical strength.^45^

Tensile strength (TS), shown in Figure 3B, remained relatively stable or slightly decreased in Ch-B samples, with no significant differences among Ch, Ch-B-10, Ch-B-20, and Ch-B-30. This outcome aligns with the structural heterogeneity observed in the SEM micrographs (Figure 2C), where discontinuities in B-containing films could act as stress concentrators and weaken matrix cohesion.^23^ On the other hand, extract-loaded films (Ch-E-10 to Ch-E-30) exhibited a statistically significant increase in TS compared to the control and biomass films, suggesting enhanced film compactness and potential intermolecular interactions between chitosan and anthocyanins at higher concentrations. The observed FTIR shifts (Figure S2), particularly in the O–H and N–H regions, confirm the formation of intermolecular hydrogen bonds between chitosan and anthocyanins. These interactions likely contributed to the enhanced compactness and integrity of the extract-loaded films, thereby explaining the observed increase in tensile strength.

The elongation at break of the films (Figure 3C) revealed a general increase in film flexibility with the addition of extract or biomass. All composite films displayed higher elongation than the control, with the effect being particularly notable in Ch-E-10, Ch-E-20, and Ch-E-30, reaching values of almost 200%. This plasticization behavior is consistent with the presence of water-soluble, low molecular weight components that enhance molecular mobility. While Ch-B-10 and Ch-B-20 also exhibited improved elongation, the effect was less pronounced in Ch-B-30, likely due to the reinforcement effect of the fiber residues.

To further investigate the interactions within the composite films, thermal properties were analyzed by TGA and DSC, with results presented in the Supporting Information (Figure S4).

TGA results revealed that the incorporation of either B or E reduced the thermal stability of the chitosan films (Figure S4A). A noticeable shift toward lower degradation onset temperatures was observed, likely due to the presence of low molecular weight compounds such as phenolics, sugars, and organic acids in the additives. These compounds may disrupt the hydrogen-bonding network of chitosan, weakening intermolecular interactions and rendering the films more thermally labile. This trend aligns with the reduction in YM observed in the mechanical tests, further supporting the plasticizing role of these additives.

Similarly, DSC analysis showed a slight decrease in glass transition temperature (*T*_g_) upon addition of B or E, confirming a reduction in matrix rigidity. As shown Figure S4B, the *T*_g_ of neat chitosan film was 30.03 °C, which decreased to 27.84 °C for Ch-E-10 and to 28.65 °C for Ch-B-10. Despite the small magnitude of change, the trend was consistent with mechanical softening observed in the films, and further illustrates the flexibility-enhancing effect of both extract and biomass.

### 3.2 Water Interaction and Barrier Properties

Given the importance of polymer–water interactions in food packaging applications, the films were evaluated in terms of moisture content, water solubility, swelling behavior, and water vapor permeability. The corresponding results are presented in Figure 4.

**Figure 4:**
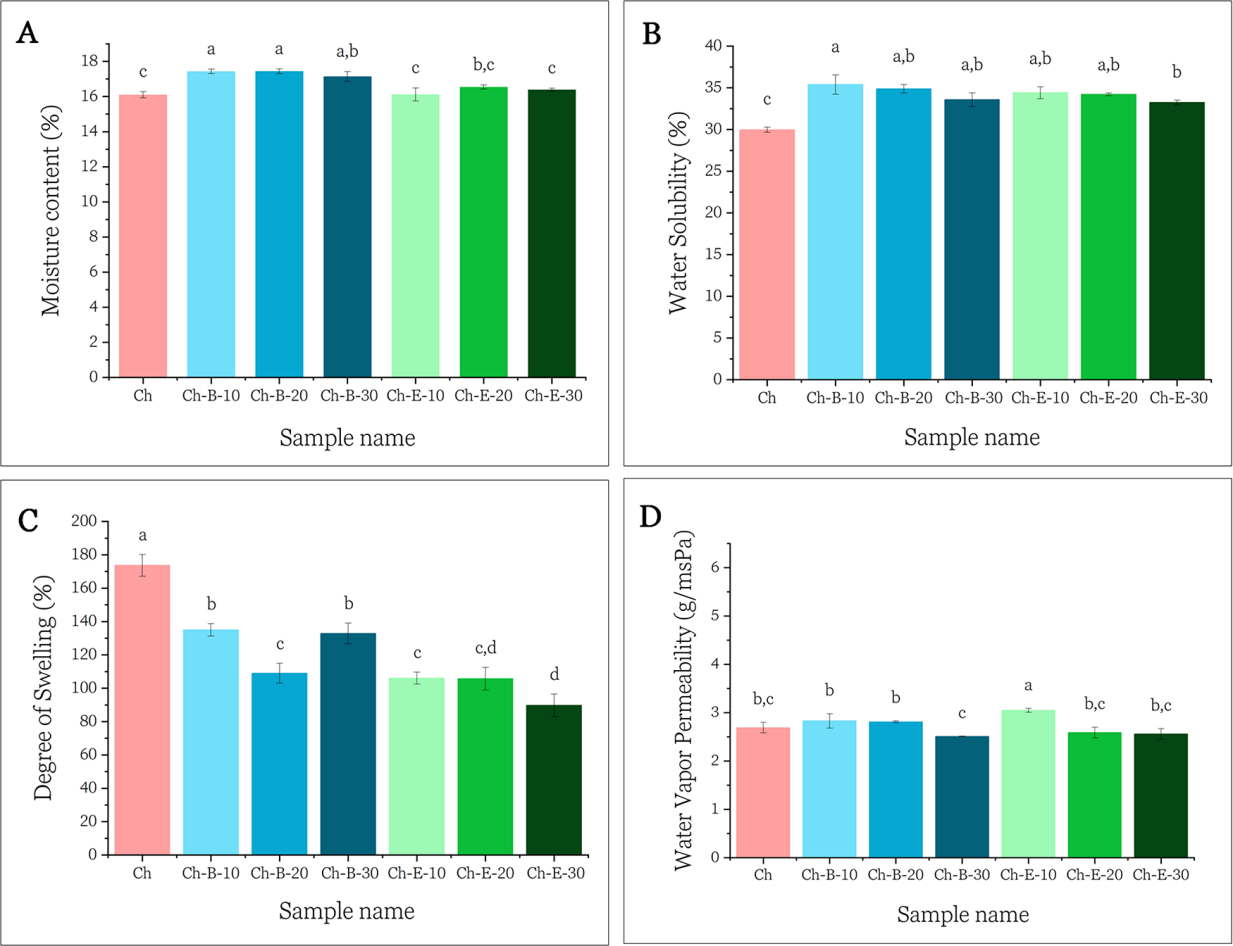
Water Interaction and barrier properties of films. (A) Moisture content (%), (B) water solubility (%), (C) degree of swelling (%), and (D) water vapor permeability (x 10^–10^ g/msPa). Different letters indicate statistically significant differences (*p* < 0.05) according to Tukey’s test.

As shown in Figure 4A, the incorporation of B slightly increased the MC of the films, while the addition of E had no significant effect. This increase in MC for B-containing films is likely due to the presence of hydrophilic constituents in the biomass, such as dietary fibers, polysaccharides, and residual sugars, which enhance water retention during film formation. Additionally, the less compact film structure observed in SEM images (Figure 2C) may have contributed to higher water uptake.

Water solubility is a key parameter for assessing the water affinity of biobased films. As shown in Figure 4B, the addition of both B and E significantly increased WS compared to the control Ch film. This effect can be attributed to the hydrophilic nature of the added components and the presence of soluble compounds such as polyphenols, sugars, and organic acids.^13^ Similar increases in WS have been reported with the incorporation of blueberry and red grape pomace extracts,^19^ citrus limetta pomace extract,^46^ olive pomace flour^47^ into chitosan matrixes and cashew nut testa extract into chitosan/poly­(vinyl alcohol) films.^48^ Furthermore, structural irregularities introduced by the pomace, as evidenced in the SEM cross sections, may create microchannels that enhance water absorption. The presence of low molecular weight solutes in B and E also likely contributes to solubility through migration during film immersion.

Interestingly, the neat chitosan film exhibited the highest swelling capacity (Figure 4C), while the incorporation of B and E significantly reduced SD values. This reduction is attributed to the partial leaching of B and E during swelling and to the lower relative content of chitosan in the composite matrix, which limits water uptake. Similar trends have been observed in chitosan films incorporated with polyphenol-rich pomace.^38,49^

The WVP of the control chitosan film was 2.69 × 10^–10^ g/msPa, in agreement with literature values: 2.39 × 10^–10^ g/msPa by Kurek et al.;^50^ 2.92 × 10^–10^ g/msPa by Sathivel et al.^51^ Films containing B and E at 10–30 wt % exhibited no significant changes in WVP (*p* > 0.05), suggesting that the incorporation of pomace did not disrupt the barrier integrity (Figure 4D). Water vapor transport is governed by several factors including the hydrophilic/hydrophobic ratio of the film components, polymer crystallinity, and the presence of microstructural defects.^52,53^

The results for WVTR are presented in the Supporting Information (Table S2). WVTR of control Ch film was 1.08 × 10^–2^ g/s m^–2^, similar to the results reported by Kurek et al.,^50^ (1.03 ± 0.05 g/s m^–2^) for their chitosan films. However, they reported lower values for WVTR with the addition of 1, 2 and 4% BBP, which could be due to the BBP concentration in the Ch films. The increase of BBP concentration (10, 20 and 30% in our case), led to an increase of the number of hydrophilic groups in the composite films, leading to greater water uptake and swelling. This increase in sorbed water facilitates the diffusion of water molecules through the polymer matrix, thereby increasing the WVTR.

### 3.3 Optical and Antioxidant Properties

In intelligent food packaging, visual appearance and oxidative stability are key to both consumer acceptance and shelf life extension. Accordingly, the optical (color and UV–vis light transmittance) and antioxidant properties of the films were assessed to evaluate their potential multifunctionality.

The optical properties of the films were assessed via UV–visible light transmittance, opacity, and pH-induced color changes, and the results are presented in Figure 5.

**Figure 5:**
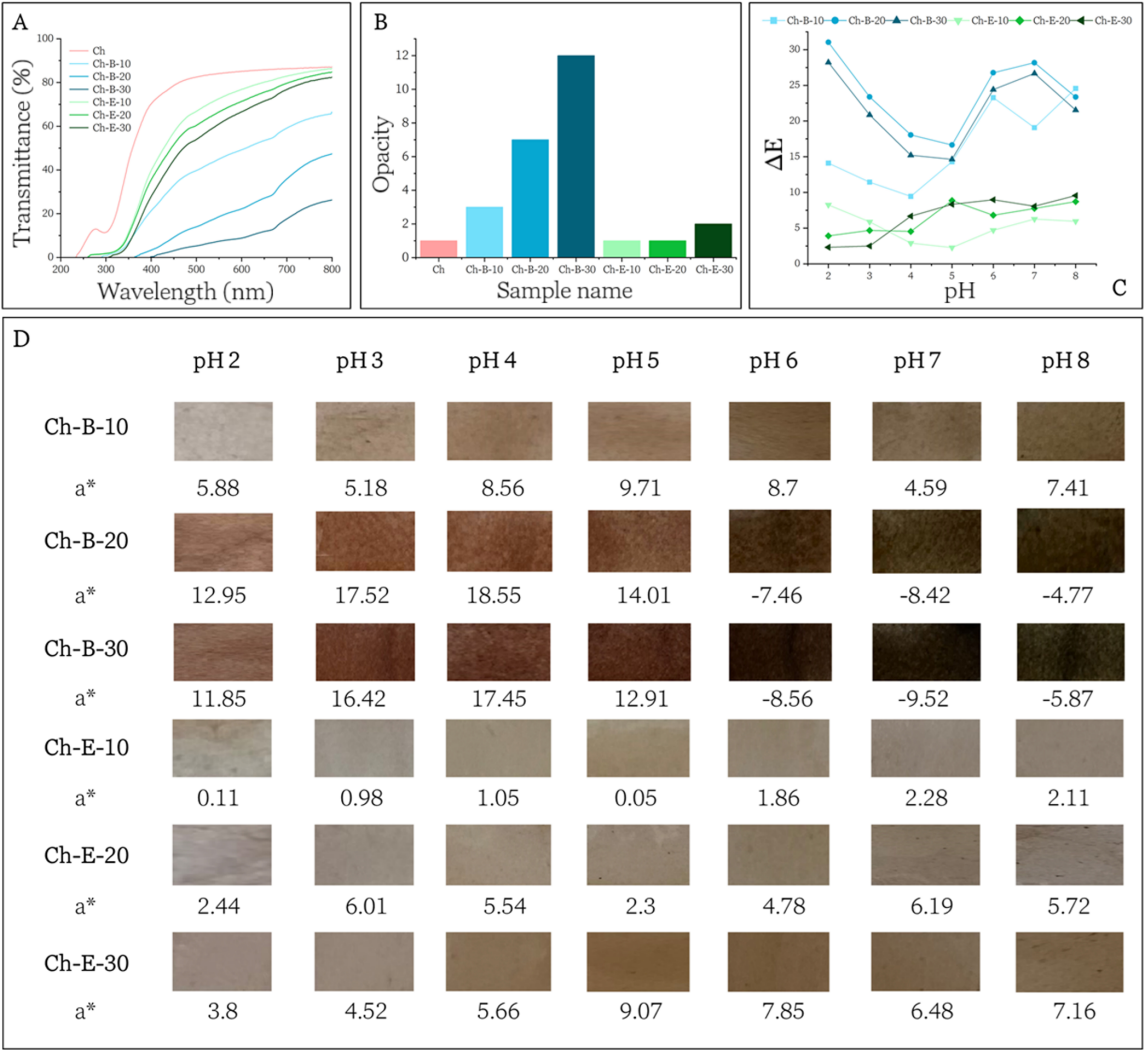
Optical properties of the films. (A) Transmittance spectra, (B) opacity, (C) Δ*E* and (D) changes of films’ color according to pH and a* parameter

Figure 5A shows the UV–vis transmittance spectra of the films in the 200–800 nm range. The control Ch film exhibited the highest transmittance across the visible spectrum, indicating its relatively high transparency. Upon incorporation of B or E, the transmittance was significantly reduced, particularly in the UV region (200–400 nm), demonstrating the films’ improved UV barrier properties. This reduction is attributed to the presence of anthocyanins and other phenolic compounds that absorb strongly in the UV range.^54^ While transparency in films is often desired for aesthetic reasons, lower transmittance is beneficial in packaging applications where protection from UV light is needed to prevent photoinduced oxidation of light-sensitive nutrients.^55^

As shown in Figure 5B, the opacity of the films increased with higher concentrations of both B and E. The Ch-B-30 sample showed the highest opacity, consistent with its dense pigmentation and greater anthocyanin content. Higher opacity is advantageous in active packaging, as it further enhances the film’s protective function against light-induced degradation.^56^ The increased opacity in B-containing films is also influenced by light scattering due to the presence of residual insoluble fiber particles.^57^

The pH sensitivity of the films was quantified through the total color difference (Δ*E*), as shown in Figure 5C. Δ*E* provides a numerical measure of visible color change between the film at each pH value and a reference condition (pH 2). Higher Δ*E* values indicate a more pronounced color shift.^34^ As seen in the graph, Ch-B-20 and Ch-B-30 films exhibited the highest Δ*E* across the pH range (especially from pH 4 to 8), confirming their strong responsiveness to pH variations. Ch-B-10 films showed moderate Δ*E* values, while Ch-E films displayed low Δ*E* values throughout, suggesting limited color reactivity. This indicates that films containing the whole biomass had a stronger and more dynamic response to pH than those containing only the anthocyanin extract, possibly due to better pigment entrapment, distribution, and protection within the composite matrix.

Complementary to these quantitative results, the visual appearance of the films immersed in buffer solutions ranging from pH 2 to 8 is presented in Figure 5D. Consistent with the Δ*E* data, Ch-B-20 and Ch-B-30 films showed the most evident and distinguishable color transitions, from light red/pink at acidic pH to dark brown or greenish tones at basic pH. These visual changes align with the typical structural transitions of anthocyanins, which shift from flavylium cations (red) at low pH to quinoidal or chalcone forms (purple/green) at higher pH.^34^

The a* parameter listed beneath each image in Figure 5D further supports these observations: values decreased markedly with increasing pH and turned negative in basic conditions, confirming a visible shift from red to green. In contrast, Ch-E films remained pale and exhibited minimal visual differences across the tested pH values, reinforcing their lower Δ*E* performance. This reduced sensitivity may be due to pigment leaching, weaker retention of anthocyanins, or less effective interaction with the film matrix.

These findings highlight that the full-biomass approach (Ch-B films) not only improves anthocyanin retention but also enhances pH responsiveness, making it more suitable for intelligent packaging applications that rely on clear and reversible visual indicators.

BBP retains a high concentration of beneficial compounds and anthocyanins, which are known for their antioxidant properties.^58^ The antioxidant properties of the films were assessed via DPPH radical scavenging activity, shown in Figure 6. All samples exhibited increasing scavenging activity over 24 h, plateauing after 12 h, suggesting sustained release of antioxidant compounds from the film matrix. The control Ch showed the lowest antioxidant activity, consistent with its lack of bioactive compounds.^37^ The addition of both B and E significantly enhanced antioxidant capacity, with a clear concentration-dependent effect. Ch-B-30 and Ch-E-30 reached the highest DPPH scavenging values (≈97–99%), indicating successful incorporation and release of phenolic antioxidants. Extract-based films showed slightly faster initial activity, while biomass-based films reached comparable levels more gradually, suggesting a more sustained release mechanism.

**Figure 6:**
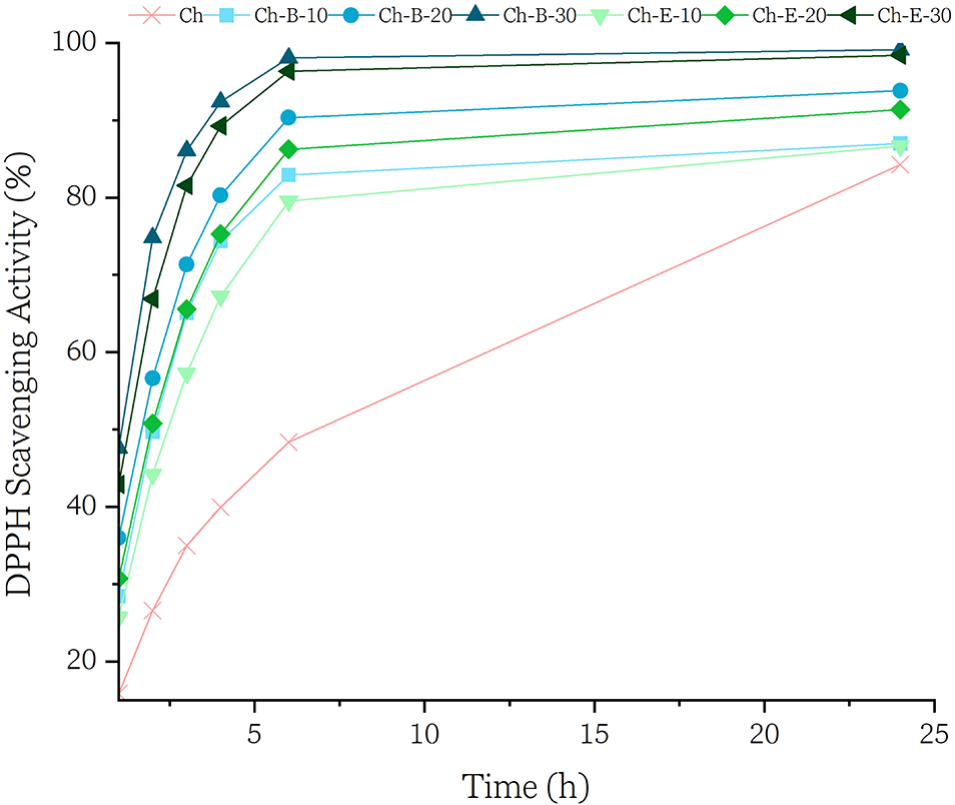
DPPH scavenging activity of films (%) as a function of time (h).

## 4 Conclusions

This study demonstrates the feasibility of developing multifunctional and environmentally friendly bioplastics through the full integration of hydrolyzed blueberry pomace into chitosan-based films. The resulting biocomposite films exhibited several performance advantages over extract-only counterparts. Notably, the inclusion of the whole biomass enhanced the films’ opacity and UV light barrier properties, while also enabling more pronounced and reversible color changes in response to pH variations. This pH-responsiveness was particularly strong in the BBP-containing films, supporting their application as intelligent indicators for freshness monitoring in food packaging. Furthermore, the sustained release of bioactive compounds from the embedded pomace contributed to high antioxidant activity, with BBP films achieving values comparable to those of extract-based films but with a slower, more prolonged release profile. The incorporation of BBP led to films with increased flexibility and adequate mechanical strength. Water vapor permeability remained stable in the biomass films, indicating that barrier functionality was preserved. Overall, the combination of functional, structural, and environmental benefits positions BBP-loaded chitosan films as promising candidates for active and intelligent food packaging. This work highlights the benefit of integrating underutilized agro-industrial byproducts into high-performance materials, offering a scalable and sustainable alternative to synthetic plastics.

## Supplementary Material



## Notes

Blueberry pomace specification; film formulations and labels; FTIR spectra of chitosan (Ch), plant extract (E), and chitosan-based composites containing the extract (Ch/E); SEM micrographs of the surface morphology of the films; thermogravimetric analysis (TGA) and differential scanning calorimetry (DSC) curves; and water vapor transmission rate (WVTR) of films (PDF)
