# Supplementary material for: Valorization of Blueberry Processing Byproducts for the Development of Intelligent Packaging: A Comparative Study on Biomass versus Extract Use in pH-Sensitive Films
Source: ACS Mater Au. 2025 Oct 27;6(1):202–12. doi: 10.1021/acsmaterialsau.5c00150 (PMC12810031; doi:10.1021/acsmaterialsau.5c00150)
Supplement: Supplementary file 1 [file mg5c00150_si_001.pdf]

SUPPORTING INFORMATION

Valorization of Blueberry Processing Byproducts for the Development of Intelligent Packaging: A Comparative Study on Biomass versus Extract Use in pH-Sensitive Films

Milica Arizanova[1,2], Elena Velickova[1], Darko Dimitrovski[1], Danila Merino[2,3*]

[1]Department of Food Technology and Biotechnology, Faculty of Technology and Metallurgy, Ss Cyril and Methodius University in Skopje, Rugjer Boskovic 16, 1000 Skopje, North Macedonia

[2]POLYMAT, Basque Center for Macromolecular Design and Engineering, University of the Basque Country UPV/EHU, Avenida de Tolosa 72, 20018 Donostia-San Sebastian, Spain.

[3]Ikerbasque, Basque Foundation for Science, 48009 Bilbao, Spain

***Corresponding author**: Dr. Danila Merino. POLYMAT, University of the Basque Country UPV/EHU, Joxe Mari Korta Center, Av. Tolosa 72, 20018, Donostia-San Sebastian, Spain. E-mail: danila.merino@ehu.eus

	EU					
	**100**	**g**	**% NRV Daily**	**6**	**g**	**% NRV Portion**
**ENERGY:**	**352**	Kcal	18%	**21**	Kcal	1%
	**1449.2**	kJ	17%	**87**	kJ	1%
**FAT:**	**16**	g	22%	**0.9**	g	1%
of which saturated fats:	1.1	g	6%	0.1	g	0%
**CARBOHYDRATES:**	**14**	g	5%	**0.8**	g	0%
of which sugars:	14	g	15%	0.8	g	1%
**FIBRE:**	**56**	g		**3.3**	g	
**PROTEIN:**	**12**	g	24%	**0.7**	g	1%
**SALT:**	**0**	g	0%	**0**	g	0%
Sodium	2.0	mg		0.1	mg	
Vitamin E	13.3	mg	111%	0.8	mg	7%

Mean values per 100g product by analysis or calculation.

**Microbiological indicators**	
Total aerobic microbial count	$<1.0*10^{5}$ cfu/ g
Yeasts	$<1.0*10^{4}$ cfu/ g
Moulds	$<1.0*10^{4}$ cfu/ g
Listeria monocytogenes	neg / 25g
Salmonella spp.	neg / 25g

Figure S1. BBP Specification

Table S1. Film formulations and labels

Components (g/1g dried film)	Formulations						
	Ch	Ch-B-10	Ch-B-20	Ch-B-30	Ch-E-10	Ch-E-20	Ch-E-30
Solution A (Ch, Glycerol, Tween 80)	1	0.9	0.8	0.7	0.9	0.8	0.7
Solution B (BBP)	-	0.1	0.2	0.3	-	-	-
Solution C (BB Extract)	-	-	-	-	0.1	0.2	0.3

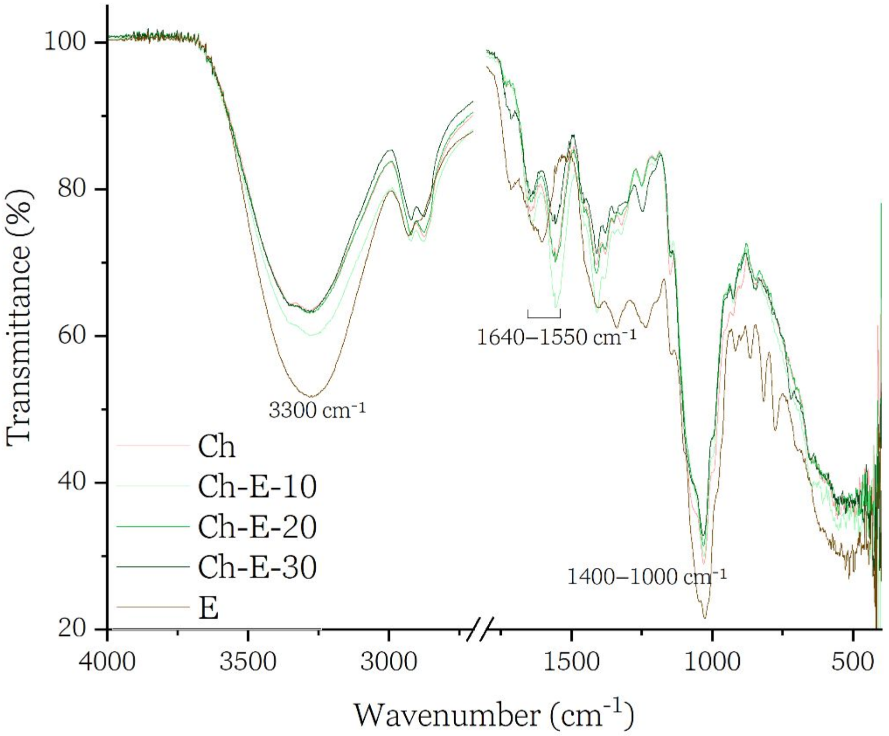

Figure S2. FTIR spectra of chitosan (Ch), plant extract (E), and chitosan-based composites containing the extract (Ch/E).

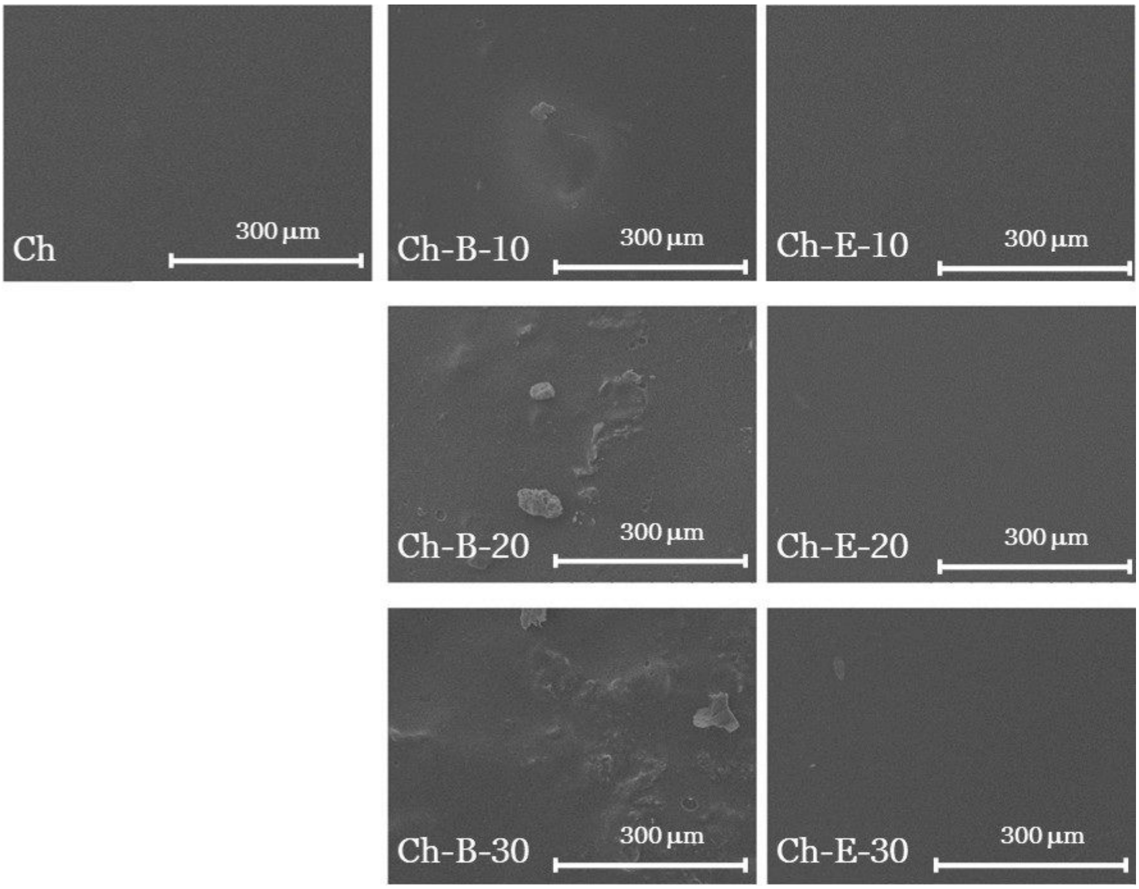

Figure S3. SEM micrographs of the surface morphology of the films: Ch, Ch-B-10, Ch-B-20, Ch-B-30, Ch-E-10, Ch-E-20, and Ch-E-30.

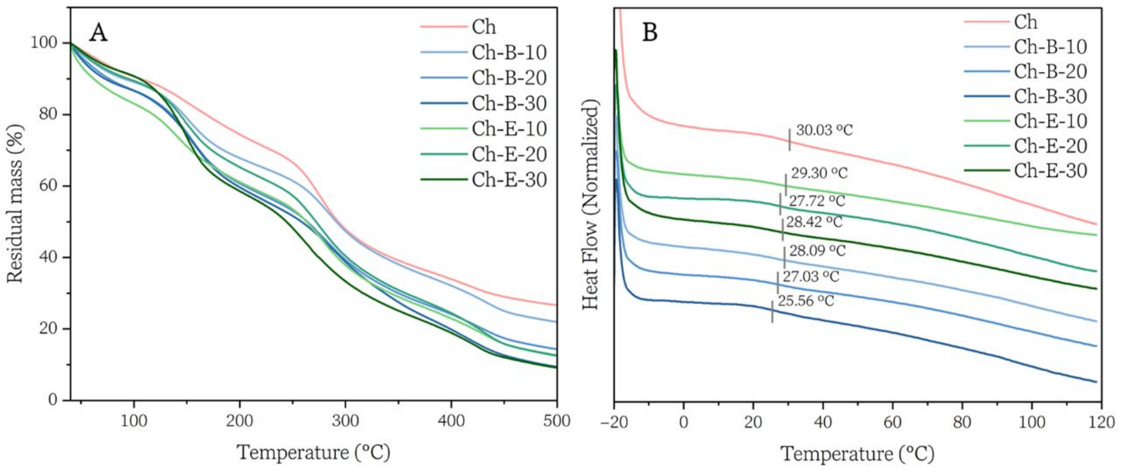

Figure S4. (**A**) Thermogravimetric analysis (TGA) and (**B**) Differential scanning calorimetry (DSC) curves of chitosan-based films.

Table S2. Water Vapor Transmission Rate (WVTR) of films

Sample	WVTR · 10^{-2} (g/s·m²)
Ch	1.08 ± 0.001^{a}
Ch-B-10	1.22 ± 0.004 ^{b}
Ch-B-20	1.50 ± 0.002 ^{c}
Ch-B-30	1.33 ± 0.001 ^{b,c}
Ch-E-10	1.24 ± 0.001 ^{b}
Ch-E-20	1.28 ± 0.003 ^{b}
Ch-E-30	1.41 ± 0.003 ^{c}
